# Artificial Intelligence in the Management of Rotator Cuff Tears

**DOI:** 10.3390/ijerph192416779

**Published:** 2022-12-14

**Authors:** Filippo Familiari, Olimpio Galasso, Federica Massazza, Michele Mercurio, Henry Fox, Uma Srikumaran, Giorgio Gasparini

**Affiliations:** 1 Department of Orthopaedic and Trauma Surgery, “Mater Domini” University Hospital, “Magna Græcia” University, 88100 Catanzaro, Italy; 2 Department of Orthopaedic Surgery, Johns Hopkins University School of Medicine, Baltimore, MD 21205, USA

**Keywords:** artificial intelligence, orthopedics, pathology, rotator cuff, shoulder, tear

## Abstract

Technological innovation is a key component of orthopedic surgery. Artificial intelligence (AI), which describes the ability of computers to process massive data and “learn” from it to produce outputs that mirror human cognition and problem solving, may become an important tool for orthopedic surgeons in the future. AI may be able to improve decision making, both clinically and surgically, via integrating additional data-driven problem solving into practice. The aim of this article will be to review the current applications of AI in the management of rotator cuff tears. The article will discuss various stages of the clinical course: predictive models and prognosis, diagnosis, intraoperative applications, and postoperative care and rehabilitation. Throughout the article, which is a review in terms of study design, we will introduce the concept of AI in rotator cuff tears and provide examples of how these tools can impact clinical practice and patient care. Though many advancements in AI have been made regarding evaluating rotator cuff tears—particularly in the realm of diagnostic imaging—further advancements are required before they become a regular facet of daily clinical practice.

## 1. Introduction

Shoulder pain has been defined as one of the most common musculoskeletal complaints managed by physicians and physical therapists [1]. The glenohumeral joint is a complex anatomic structure commonly affected by injury such as tendinopathy and rotator cuff tears (RCTs) [2]. RCTs are one of the most common shoulder injuries, affecting more than 40% of patients over the age of 60 and resulting in 30,000–75,000 surgical repairs performed annually [3]. RCT symptoms, such as pain, loss of motion, and weakness, predominantly affect active individuals and can negatively impact people’s daily activities thus leading to an overall poor quality-of-life experience. The functional disability of rotator cuff injuries affects not just the physical aspects of a patient’s life but also the mental and social aspects [1]. Disruption of the force balance and normal shoulder stability and motion after loss of tendon function initiates changes in almost all adjacent tissues [4]. Despite improvements in our understanding of this disease process and advances in surgical treatment, healing after rotator cuff repair remains a significant clinical challenge [3,5]. There remains a need for innovative repair strategies that augment the repair by mechanically reinforcing it, while simultaneously biologically enhancing the intrinsic healing potential of the tendon [6].

Within the past several decades, technologies based on artificial intelligence (AI) have helped face many new challenges in medicine [7]. AI refers to a branch of computer systems able to execute tasks that imitate human cognitive functions, such as learning and problem-solving, by analysing and comparing data [8,9]. The aim of AI and machine learning (ML), defined as the scientific discipline that focuses on how computers learn from data, ref. [10] is to extract relevant information from massive healthcare data and to assist clinical decision-making. By refining decision making, medical errors can be minimized, clinical outcomes can be maximized, and the overall quality and efficiency of care delivery can be enhanced [11].

**Figure 1:**
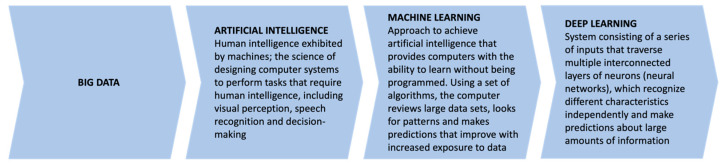
This flowsheet defines and clarifies relevant terms and concepts used throughout the article. “Big Data” can be applied to multiple processes that are within the realm of artificial intelligence. Machine learning and deep learning are subsets of artificial intelligence, and each process uses “big data” as inputs.

A large amount of structured information can be analysed through ML techniques, which can be combined with various analytical algorithms to create forecast models on input variables [10] (Figure 1).

Moreover, deep learning (DL), a sub-category of AI, should be mentioned due to its wide application in healthcare. This system consists of a series of inputs that traverse multiple interconnected layers of neurons (neural networks), which recognize different characteristics independently and make predictions about large amounts of information [10].

The greatest strength of many AI algorithms is that they are set to learn from data without human intervention. This ability offers also exciting possibilities for musculoskeletal radiology [12]. 

Recently, many studies have explored the role of AI and its subgroups in the orthopaedic field. AI offers a potential avenue to augment the orthopaedic surgeon while also maximizing value in the delivery of care [13]. One specific area of investigation is how AI can contribute to the accurate diagnosis and characterization of RCTs with imaging studies. Amidst several imaging studies, up to now, magnetic resonance imaging (MRI) and MR arthrography are the most accurate non-invasive tests used to define shoulder pathologies such as RCTs. MRI contributes information on several cuff-related factors that influence the success of surgical repair, such as tear size (anteroposterior tear length, mediolateral tear length, and tear size area), tear depth, tendon quality, tendon fibre retraction, fatty infiltration of the rotator cuff muscles, and the number of torn tendons [12]. Liu et al. [14] performed a network meta-analysis of 144 diagnostic studies and determined that the diagnostic performances of MRI and MR arthrography for diagnosing RCTs yielded pooled sensitivities of 80–87% and specificities of 81–90%.

## 2. Materials and Methods

The aim of this study is to review the current applications of AI in the orthopaedic field for rotator cuff pathologies across the spectrum of care, from diagnosis to the operating room (OR) and subsequent recovery. Herein we will discuss how AI applies to predictive models, diagnosis, intraoperative management, and postoperative care and rehabilitation. To our knowledge, this is the first review paper to offer a summative overview of how artificial intelligence has been applied to multiple aspects of rotator cuff care. 

A review of the literature was undertaken using the PubMed database. Keywords included “rotator cuff” and “artificial learning”, as well as “deep learning”. Journal articles from the past ten years were considered. Abstracts were reviewed for relevancy, and relevant articles (which discussed AI in the context of rotator cuff tears) were selected for full text review. Full text review included analysis of topic of study, study design and methods, and extraction of key results. The articles selected are reflected in Table 1. 

## 3. Results

In our results section, we consider the applications of AI and Deep Learning to rotator cuff treatment in the following subsections: (A) Diagnosis, (B) Postoperative care and rehabilitation, and (C) Challenges and future perspectives.

A. Diagnosis

A precise diagnosis is pivotal to planning and performing a successful RCT repair. Beyond physical examination, this can be achieved through various imaging techniques. To date, the best non-invasive imaging techniques are ultrasonography imaging (US) and MRI [14,15,16,17]. Computed tomography (CT) and X-rays are also useful tools for the study of shoulder pathologies. AI has been applied to multiple imaging modalities to diagnose and characterize RCTs more accurately. This section briefly discusses the role of AI in X-ray and CT interpretation. The role of AI in refining MRI for diagnosing RCTs is then explored in detail, followed by a discussion of AI applications in ultrasound. 

X-rays have been considered to hold a high negative predictive value (NPV) in the diagnosis of RCTs [18]. An algorithm has been developed to confidently rule out RCTs based on conventional shoulder X-rays in patients clinically suspected of having a RCT [18]. The deep learning algorithm was trained with only three views (true anteroposterior, caudal 30° tilt, and supraspinatus outlet) which are known to play a helpful role in predicting RCT [19,20,21,22]. The ML model showed a sensitivity of 97.3%, a negative likelihood of 0.06 and a NPV of 96.6%, higher in patients with age < 60 years [18].

Artificial intelligence algorithms for diagnosing RCTs with CT scans have also been developed. Taghizadeh et al. [23] conducted a study aimed at developing a convolutional neural network (CNN) which would be able to automatically quantify and characterize the level of degeneration of rotator cuff muscles from shoulder CT images. The assessment included factors such as muscle atrophy and fatty infiltration, important parameters in surgical decision-making and overall patient management [24]. The CNN was developed using standardized sagittal-oblique view CT. It provided a “good” or “very good” approach in estimating (1) muscle atrophy, (2) fatty infiltration, and (3) overall muscle degeneration. However, the CNN was not able to calculate the secondary bone formation as well as the human rater did. The proposed algorithm was able to determine the premorbid locations, shapes, and boundaries of all four rotator cuff muscles with an accuracy comparable with manual segmentations; thus, demonstrating that it could be applied in clinical practice.

In contrast to CT and X-ray, MRI is the predominant imaging study used for the diagnosis of RCTs due to the ability to precisely assess fatty infiltration and/or muscle atrophy. Initial studies which applied AI to MRIs for shoulder pathology examined the performance of software which could successfully filter out the MRI signals that are “noisy,” thus applying pattern recognition in the interpretation of each MRI [25]. The authors affirmed that applying this technique would reduce the dimensions of the input vector, the computing time, and the data storage required, while simultaneously improving the diagnostic performance. 

More recently, authors have studied how deep learning (DL) can detect supraspinatus tearing on MRI [26]. The authors defined an algorithm for automatic detection of supraspinatus tears on MRI based on T2-weighted coronal oblique images, improving their model by adding Patte score [27] and Ellman score [28] to define the grade of the tear. A segmentation network designed as a transfer-learning approach (in which all models were trained using a step-based learning rate reduction strategy and automatically stopped at a plateau in the training loss) was proposed. This model was able to improve clinical communication and optimize management decisions.

Additional studies have focused on applying AI to T1-weighted sequences rather than T2-weighted ones. Authors developed and validated CNN method capable of both selecting a specific shoulder sagittal MR image (Y-view) and automatically segmenting rotator cuff (RC) muscles [29]. With the use of two models (Model A: Y-view selection and Model B: muscle segmentation) the authors were able to demonstrate that a CNN classification method could accurately select an appropriate shoulder Y-view and accurately segment multiple RC muscles at that level. 

Just recently, another study analysed the use of deep-learning framework to study occupation ratio and fatty infiltration of the supraspinatus muscle [30]. Scapular sagittal Y-view MRI images (T1-weighted) were used as baseline for obtaining reliable indicators of the supraspinatus muscle status. Manually measuring the occupation ratio and fatty infiltration is time intensive; the algorithm proposed in the paper demonstrated that the CNN exhibited excellent agreement with clinicians, resulting in an efficient and accurate segmentation of the supraspinatus muscle and fossa. This success enabled physicians to automatically calculate the occupation ratio [31]. 

Investigators have also explored whether AI can analyse MRIs to predict the reparability of massive RCTs [32]. The severity of disease was defined based on Goutallier grade [33] and occupation ratio [31]. This algorithm confirmed that while it is possible to detect the occupation ratio by DL, the detection accuracy of the fossa region according to severity did not show satisfying results. The authors suggested that the development of a 3D-CNN, capable of analysing the neighbouring slice, would represent a future step towards creating a more accurate algorithm [32]. Accordingly, a 3D-CNN to classify RCTs was introduced [34]; with the use of the class activation map (CAM) method [35], this system could visualize the location and size of RCT in 3D-MRI with a very simple processing of training data [34].

An additional field of study with AI is how to decrease MRI scan time while improving image quality. A system has been described which overcomes the trade-off between scan time and image quality, providing high-fidelity images with reduced noise levels [36]. To define the ability of this new system, the authors used sets of sequences including an axial intermediate-weighted fat-suppressed FSE sequence, an oblique coronal T2-weighted fat-suppressed FSE sequence, and an oblique sagittal T2-weighted fat-suppressed FSE sequence. They analysed the three series of MRI and found that the use of deep learning-based reconstruction (DLR) with accelerated sequences resulted in improved image quality and low artifacts compared with accelerated sequences without DLR. Moreover, the authors stated that DLR was able to substantially reduce shoulder MRI scan times by facilitating the clinical application of accelerated sequences. 

US has been identified as a valid alternative to MRI [15]. Ultrasound offers several advantages over MRI including real-time dynamic capture, wide availability, low-cost, and time efficiency [37]. Authors have designed a model whereby deep learning assists with segmentation of the RCT on ultrasound. This was a (CNN)-based DL architecture (the SMART-CA), to perform an accurate segmentation of RCT in US [38]. This model was designed to use the pre-trained architecture to extract specific feature maps related to RCT. The SMART-CA consists of three key components: (1) a pre-trained encoder, (2) a trainable encoder, and (3) a decoder. The variables in the trainable encoder are optimized to accurately predict the RCT. This study demonstrated that the proposed SMART-CA, pre-trained through a classification task which predicts the presence of an RCT in ultrasound images, outperforms the current model in the accurate segmentation of RCTs in US. Aside from conventional US, 3D quantitative analysis of RCT regions (and especially 3D volumetric segmentation done by 3D US) is crucial to precisely estimate the tear configuration and decide surgical planning. Ultrasonic image segmentation enables delineation of the boundaries of an RCT on an ultrasound image [39]. This protocol may lead to changes in RCT diagnosis.

B. Postoperative Care and Rehabilitation

Conservative management with physical therapy has been established as an effective first line of treatment for RTCs. Participation in a physical therapy program is considered one of the greatest predictors of successful conservative management of these lesions [40]; however, adherence to the physical therapy protocols remains very low (around 50%) [41]. Progress with physical therapy is often slow-going and incremental and demands significant time and effort on behalf of patients. This can oftentimes become draining and/or frustrating for patients who do not have the proper expectations. 

To prove the technical feasibility of a smartwatch device and supervised machine learning (ML) approach to monitor and assess the at-home adherence of shoulder physiotherapy exercise protocols, several studies used inertial sensors for upper extremity motion tracking of shoulder kinematic analysis and activity recognition [42]. The tracker used was an Apple Watch provided by a six-axis (acceleration and gyroscope) inertial sensor. Using the extrapolated data, an algorithm was developed that could measure the frequency and duration of each prescribed exercise. This model, a convolutional recurrent neural network (CRNN) with approximately 300,000 parameters, was still sufficiently small for real-time operation on a smart watch, thus suitable for daily life tracking. This study marked an important step towards objective measurement of adherence to at-home shoulder physiotherapy exercise protocols. It should be considered the new frontier for both pre-rehabilitation and post-surgical rehabilitation.

C. Challenges and Future Perspectives

Despite the promising results of AI implementation in orthopaedic surgery and radiology to date, this technology has yet to be widely accepted and implemented in the clinical realm. Challenges and pitfalls can accompany the development and deployment of AI systems in a diagnostic imaging environment. As reported by Jared Dunnmon in “Separating hope from hype”, AI has yet to find a consistently defined and clinically relevant role in radiology or orthopaedic care of the rotator cuff [43]. To that end, AI in the operative management of rotator cuff care has yet to be employed. While this possibility will take years of research and develop to potentially manifest, perhaps in the future, surgeons will use AI-driven data to optimize cuff repairs intra-operatively, for example, using a heads-up display (while scrubbed in) to allow for AI-generated guidance on where to repair the cuff. Another theoretical application would be AI-driven feedback (during arthroscopy) on whether a cuff is repairable, or what the probabilities of subsequent repair failure may be. While these are exciting frontiers to consider, the intra-operative application of AI in rotator cuff repair is likely years in the future. 

No subject is accurately discussed without consideration of limitations. Limitations of AI in rotator cuff care include the fact that these areas of research are nascent, not yet within the clinical realm, and require further validation/external generalizability before they are adopted in mainstream clinical care (for example, using AI in imaging). Another limitation is that AI technologies may prove disproportionately expensive upon introduction. AI will also be met with scepticism from some surgeons and patients, given that it would represent significantly new technology. It would also lay the debate for whether experienced surgeons or AI is more dependable. Limitations of our study include its retrospective review nature, and restriction of our literature search to one database.

The widespread application of AI has opened new debates about legislative issues and the protection of privacy. While AI holds promise to improve care, the massive volume of personal health information that it would process could put personalized health information at risk of privacy breaches. As with other matters related to personal health information, a delicate balance exists between progress and personal privacy. As AI research and applications expand, healthcare regulations and legislative actions will likely be necessary. If machine learning and AI becomes more available clinically, efforts will need to be made to equally provide access for maximal benefit worldwide. If AI were to only be available in specific geographic areas, it would likely worsen the already-present geographic health disparities. Future implementation of AI also represents a challenge. Healthcare organizations will have to invest in training and support infrastructure, in order for healthcare professionals to feel comfortable with applying these new tools [44].

## 4. Conclusions

Herein, we have discussed current applications of AI in the orthopaedic field for diagnosis and treatment of rotator cuff tears. While a growing body of studies have investigated the applicability of AI to rotator cuff tear management—particularly in applying AI to shoulder MRI interpretation—these applications are still primarily limited to the experimental realm. In the future, AI will likely play an impactful role in delivering accurate, efficient, and high-quality care to patients with rotator cuff tears. 

## Figures and Tables

**Table 1:** Characteristics of studies on artificial intelligence in rotator cuff tear.

Author	Journal	Title	Year of Pubblication	Where	Imaging	Pathology
Chen-Chiang LIN, Chih-Nan Wang, Yang-Kun Ou, Jachih Fu	Japanese society for magnetic resonance in medicine	Combined image enhancement, feature extraction, and classification protocol to improve detection and diagnosis of rotator-cuff tear on MR imaging	2014	Taiwan	MRI	Rotator-cuff tear
David M. Burns MD, Nathan Leung, Michael Hardisty PhD, Cari Whyne PhD, Patrick Henry MD FRCSC PhD, and Stewart McLachlin PhD	IPEM—institute of physics and engineering in medicine	Shoulder Physiotherapy Exercise Recognition: Machine Learning the Inertial Signals from a Smartwatch	2018	Canada	Smartwatch	Shoulder
Elham Taghizadeh & Oskar Truffer & Fabio Becce & Sylvain Eminian & Stacey Gidoin & Alexandre Terrier & Alain Farron & Philippe Buchle	Springer—European Radiology	Deep learning for the rapid automatic quantification and characterization of rotator cuff muscle degeneration from shoulder CT datasets	2020	Switzerland	CT	Rotator cuff muscle degeneration
Eungjune Shim, Joon Yub Kim, Jong Pil Yoon, Se-Young Ki, Taewoo Lho, Youngjun Kim & Seok Won Chung	Nature: scientific reports	Automated rotator cuff tear classification using 3D convolutional neural network	2020	Korea	MRI	Rotator cuff Tear
Evangelia E. Vassalou; Michail E. Klontzas; Kostas Marias; Apostolos H. Karantanas	Springer—skeletal radiology	Predicting long-term outcomes of ultrasound-guided percutaneous irrigation of calcific tendinopathy with the use of machine learning	2021	Greece	ultrasound-guided	Calcific tendinopathy
Giovanna Medina & Colleen G. Buckless & Eamon Thomasson & Luke S. Oh & Martin Torriani	Springer—skeletal radiology	Deep learning method for segmentation of rotator cuff muscles on MR images	2020	USA	MRI	Segmentation of rotator cuff muscles
Jason Yao; Leonid Chepelev; Yashmin Nisha; Paul Sathiadoss; Frank J. Rybicki; Adnan M. Sheikh	Springer—skeletal radiology	Evaluation of a deep learning method for the automated detection of supraspinatus tears on MRI	2022	Canada-USA	MRI	Supraspinatus tears
Joo Young Kim, Kyunghan Ro, Sungmin You, Bo Rum Nam, Sunhyun Yook, Hee Seol Park, Jae Chul Yoo, Eunkyoung Park, Kyeongwon Cho, Baek Hwan Cho, In Young Kim	ELSEVIER—Computer Methods and Programs in Biomedicine	Development of an automatic muscle atrophy measuring algorithm to calculate the ratio of supraspinatus in supraspinous fossa using deep learning	2019	Korea	MRI	Muscle atrophy
Kyungsu Lee, Jun Young Kim, Moon Hwan Lee, Chang-Hyuk Choi and Jae Youn Hwang	Sensors	Imbalanced Loss-Integrated Deep-Learning-Based Ultrasound Image Analysis for Diagnosis of Rotator-Cuff Tear	2021	Switzerland	Ultrasound	Rotator-cuff tear
Kyunghan Ro, Joo Young Kim, Heeseol Park, Baek Hwan Cho, In Young Kim, Seung Bo Shim, In Young Choi & Jae Chul Yoo	Nature: scientific reports	Deep-learning framework and computer assisted fatty infiltration analysis for the supraspinatus muscle in MRI	2021	Korea	MRI	Supraspinatus muscle
Seok Hahn, MD, Jisook Yi, MD, Ho-Joon Lee, MD, Yedaun Lee, MD, Yun-Jung Lim, MD, Jin-Young Bang, MD, Hyunwoong Kim, MS, Joonsung Lee, PhD	Ajronline	Image Quality and Diagnostic Performance of Accelerated Shoulder MRI With Deep Learning–Based Reconstruction	2021	Korea	MRI	Shoulder
Shengtao Dong, Jie Li, Haozong Zhao, Yuanyuan Zheng, Yaoning Chen, Junxi Shen, Hua Yang, and Jieyang Zhu	Hindawi Computational Intelligence and Neuroscience	Risk Factor Analysis for Predicting the Onset of Rotator Cuff Calcific Tendinitis Based on Artificial Intelligence	2022	China	RX	Rotator cuff calcific tendinitis
Youngjune Kim & Dongjun Choi & Kyong Joon Lee & Yusuhn Kang & Joong Mo Ahn & Eugene Lee & Joon Woo Lee & Heung Sik Kang	Springer—European Radiology	Ruling out rotator cuff tear in shoulder radiograph series using deep learning: redefining the role of conventional radiograph	2019	South Korea	radiograph	Rotator cuff tear

## Data Availability

Not applicable.
